# RASCL: RAPID ASSESSMENT OF SARS-COV-2 CLADES THROUGH MOLECULAR SEQUENCE ANALYSIS

**DOI:** 10.1101/2022.01.15.476448

**Published:** 2022-01-18

**Authors:** Alexander G Lucaci, Jordan D Zehr, Stephen D Shank, Dave Bouvier, Han Mei, Anton Nekrutenko, Darren P Martin, Sergei L Kosakovsky Pond

**Affiliations:** 1 Institute for Genomics and Evolutionary Medicine, Temple University, Philadelphia, Pennsylvania, USA; 2 Department of Biochemistry and Molecular Biology, The Pennsylvania State University, University Park, PA, USA; 3 Institute of Infectious Diseases and Molecular Medicine, Division Of Computational Biology, Department of Integrative Biomedical Sciences, University of Cape Town, Cape Town 7701, South Africa

## Abstract

An important component of efforts to manage the ongoing COVID19 pandemic is the Rapid Assessment of how natural selection contributes to the emergence and proliferation of potentially dangerous SARS-CoV-2 lineages and CLades (RASCL). The RASCL pipeline enables continuous comparative phylogenetics-based selection analyses of rapidly growing clade-focused genome surveillance datasets, such as those produced following the initial detection of potentially dangerous variants. From such datasets RASCL automatically generates down-sampled codon alignments of individual genes/ORFs containing contextualizing background reference sequences, analyzes these with a battery of selection tests, and outputs results as both machine readable JSON files, and interactive notebook-based visualizations.

Rapid characterization and assessment of the clade-specific molecular features of individual persistent or rapidly expanding SARS-CoV-2 lineages has become an important component of efforts to monitor and manage the COVID19 pandemic. Analyses of natural selection have been broadly incorporated into such assessments as a primary tool for inferring the selective processes under which novel SARS-CoV-2 variants evolve (Tegally *et al.,* 2021, Faria *et al.,* 2021, D. Martin *et al.,* 2021, MacLean et al., 2021). Ongoing monitoring of emergent variants of interest (VOI) or concern (VOC) can detect potentially adaptive mutations before they rise to high frequency, and help establish the relationships between individual mutations and key viral characteristics including pathogenicity, transmissibility, and drug resistance (Hamed *et al*., 2021, Young *et al*., 2021, Luchsinger *et al*., 2021, Abdool *et.al*., 2021, Cyrus Maher *et al.,* 2021). Molecular patterns of ongoing selection that are evident within sequences sampled from particular VOI or VOC clades may also reveal the sub-lineages within these clades that carry potentially fitness-enhancing mutations and which are therefore most likely to drive future viral transmission (Rambaut *et al*., 2020).

Here, we present RASCL (Rapid Assessment of SARS-CoV-2 CLades), an analytic pipeline designed to investigate the nature and extent of selective forces acting on viral genes in SARS-CoV-2 sequences through comparative phylogenetic analyses (Figure 1A). RASCL is implemented as an easy-to-use, standalone pipeline and as a web application, integrated in the Galaxy framework and available for use on powerful public computing infrastructure (Afgan *et al.,* 2018).

The RASCL pipeline takes as input (i) a “query” dataset comprising a single FASTA file containing unaligned SARS-CoV-2 full or partial genomes belonging to a clade of interest (e.g., all sequences from the PANGO lineage, B.1.617.2) and (ii) a generic “background” dataset that might comprise, for example, a set of sequences that are representative of global SARS-CoV-2 genomic diversity assembled from ViPR (Pickett *et al.,* 2012). It is not necessary to remove sequences in the query dataset from the reference dataset -- the pipeline will do this automatically. The choice of “query” and “background” datasets is analysis-specific. For example, if another clade of interest is provided as background it is possible to identify sites that are evolving differently between two clades directly. Other sensible choices of query sequences might be: sequences from a specific country/region, or sequences sampled during a particular time period. Following the disassembly of whole genome datasets into individual coding sequences (based on the NCBI SARS-CoV-2 reference annotation), the gene datasets (each containing a set of query and background sequences) are processed in parallel.

Using complete linkage distance clustering implemented in the TN93 package (https://github.com/veg/tn93), RASCL subsamples from available sequences while attempting to maintain genomic diversity; the clustering threshold distance is chosen automatically to include no more than a user-specified number of genomes (e.g., 300). A combined (query and background) alignment is created with only the sequences that are divergent enough to be useful for subsequent selection analyses being retained from the background dataset. Inference of a maximum likelihood phylogenetic tree (RAxML-NG, Kozlov *et al*., 2019, or IQ-TREE, Nguyen *et al*., 2015) is performed on the combined dataset and the query and background branches of this tree are labeled. Selection analyses are then performed with state of the art molecular evolution models implemented in HyPhy (Pond *et al.,* 2020).

1. SLAC: performs substitution mapping (Pond and Frost, 2005)
2. BGM: identifies groups of sites that are apparently co-evolving (Poon *et al*., 2008)
3. FEL: locates codon sites with evidence of pervasive positive diversifying or negative selection (Pond and Frost, 2005),
4. MEME: locates codon sites with evidence of episodic positive diversifying selection, (Murrell *et al*., 2012)
5. BUSTEDS: tests for gene-wide episodic selection (Wisotsky *et al*., 2020)
6. RELAX: compare gene-wide selection pressure between the query clade and background sequences (Wertheim *et al*., 2015),
7. CFEL: comparison site-by-site selection pressure between query and background sequences (Pond *et al*., 2021).
8. FADE: identify amino-acid sites with evidence of directional selection (Pond et.al., 2008)

To mitigate the potentially confounding influences of within-host evolution and sequencing errors, these analyses are performed only on internal branches of the phylogenetic tree (Lorenzo-Redondo *et al.,* 2016). Results are combined into two machine readable JSON files (“Summary” and “Annotation”) that are used for web processing. A feature-rich interactive notebook in ObservableHQ (Perkel 2021, https://observablehq.com/@aglucaci/rascl) is used to visualize and summarize RASCL results (Figure 1B)

RASCL is currently available in two distributions:(i) through a web interface via the Galaxy Project as a workflow (https://usegalaxy.eu/u/hyphy/w/rascl); and (ii) as a standalone pipeline via a dedicated GitHub (https://github.com/veg/RASCL) repository. For the web application implementation, the alignment, tree and analysis results are stored and made web-accessible via the Galaxy platform. Results are visualized with an interactive notebook hosted on ObservableHQ (Figure 1B; Perkel 2021) that includes an alignment viewer, a visualization of individual codons/amino acid states at user-selected sites mapped onto the tips of a phylogenetic tree, and detailed tabulated information on analysis results for individual genes and codon-sites.

RASCL has been used to characterize the role of natural selection in the emergence of the Beta (Tegally *et al.,* 2021), Gamma (Faria *et al.,* 2021), and Omicron (Moyo *et al.,*2021) VOC lineages, and for identifying patterns of convergent evolution in N501Y SARS-CoV-2 lineages (Martin et al., 2021). Whenever future genomic surveillance efforts reveal new potentially problematic SARS-CoV-2 lineages, we anticipate that RASCL will be productively used to analyze these too. Finally, RASCL has been designed so that, with minimal modification, it can also be adapted to analyze any other viral pathogens for which sufficient sequencing data is available.

## Figures and Tables

**Figure 1.**
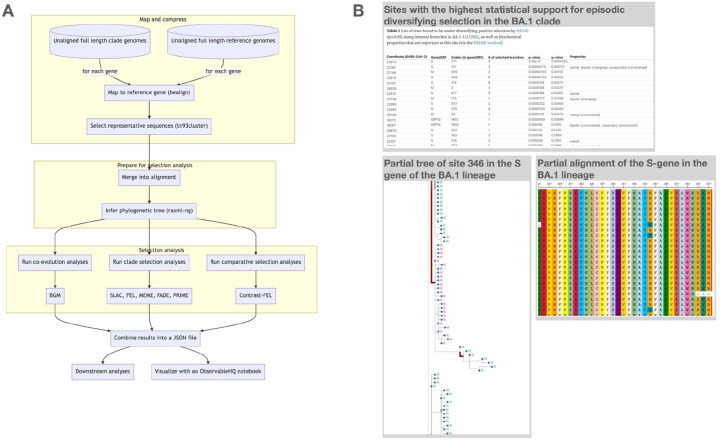
(A) A flowchart diagram of the main analytic engine of RASCL. (B) Examples of the ObservableHQ visualization notebook elements for the main Omicron clade (BA.1).
